# Prediction and associated factors of non-steroidal anti-inflammatory drugs efficacy in migraine treatment

**DOI:** 10.3389/fphar.2022.1002080

**Published:** 2022-11-18

**Authors:** Zhao-Xuan Lu, Bing-Qing Dong, Heng-Le Wei, Liang Chen

**Affiliations:** ^1^ Department of Interventional and Vascular Radiology, Nanjing First Hospital, Nanjing Medical University, Nanjing, China; ^2^ Department of Radiology, Kunshan Hospital of Traditional Chinese Medicine, Kunshan, China; ^**3**^ Department of Radiology, The Affiliated Jiangning Hospital with Nanjing Medical University, Nanjing, Jiangsu, China

**Keywords:** migraine, multivariable logistic regression, machine learning, efficacy, non-steroidal anti-inflammatory drugs

## Abstract

**Background:** The selection strategy of non-steroidal anti-inflammatory drugs (NSAIDs) for migraine is hard to judge whether it is effective, leading to unnecessary exposure to insufficient or lengthy treatment trials. The goal of the study was to investigate potential predictors of NSAIDs efficacy in migraine therapy and to explore their influence on efficacy.

**Methods:** 610 migraine patients were recruited and assigned into responders and non-responders. Potential predictors among demographic and clinical characteristics for NSAIDs efficacy were extracted using multivariable logistic regression (LR) analysis, and were applied to construct prediction models *via* machine learning (ML) algorithms. Finally, Cochran-Mantel-Haenszel tests were used to examine the impact of each predictor on drug efficacy.

**Results:** Multivariate LR analysis revealed migraine-related (disease duration, headache intensity and frequency) and psychiatric (anxiety, depression and sleep disorder) characteristics were predictive of NSAIDs efficacy. The accuracies of ML models using support vector machine, decision tree and multilayer perceptron were 0.712, 0.741, and 0.715, respectively. Cochran-Mantel-Haenszel test showed that, for variables with homogeneity of odds ratio, disease duration, frequency, anxiety, and depression and sleep disorder were associated with decreased likelihood of response to all NSAIDs. However, the variabilities in the efficacy of acetaminophen and celecoxib between patients with mild and severe headache intensity were not confirmed.

**Conclusion:** Migraine-related and psychiatric parameters play a critical role in predicting the outcomes of acute migraine treatment. These models based on predictors could optimize drug selection and improve benefits from the start of treatment.

## 1 Introduction

Migraine is a primary neurological disease, that is, characterized by recurrent episodes of debilitating moderate-severe headache. It is highly prevalent in the global population and profoundly affects patients mentally and physically (Dodick, 2018). Evidence-based as well as consensus-based clinical practice guidelines recommend multiple analgesic medications including triptans, nonsteroidal anti-inflammatory drugs (NSAIDs), calcitonin gene-related peptide (CGRP) receptor antagonists and serotonin agonists (Ailani et al., 2021; Kouremenos et al., 2019; Sacco et al., 2022). Currently, NSAIDs are the most commonly used analgesic agents worldwide (Orlova et al., 2022). Although NSAIDs have been recommended as first-line drugs (Ashina, 2020), a notable proportion of patients with migraine do not respond to analgesic medications, contributing to a large burden of migraine-related disability (Schulte and May 2015). Moreover, the NSAIDs are associated with a higher risk of side effects such as gastrointestinal bleeding and liver damage (Bindu et al., 2020). All these undoubtedly degrade the quality of life, increase personal costs and exacerbate the migraine-related burden on society. Therefore, there is an urgent need to identify or develop effective therapy for migraine.

Machine learning (ML) technology offers a potential solution to this problem. The advantage of ML is that it does not require any assumptions about the input variables and their relationships with the output. In addition, it is a fully data-driven learning method without rules-based programming. Currently, ML has been widely applied in the diagnosis and prognosis of various diseases and has shown encouraging results (Gou et al., 2022; Palaniyappan et al., 2019; Zhang et al., 2019). In the field of headache, these approaches have been used for neuroimaging analysis. Some ML models were generated from neuroimaging data, and used to classify migraine subtypes (Chong et al., 2017), clinical features (Mu et al., 2020), and efficacy of acupuncture treatment (Yang et al., 2020; Yin et al., 2020). A ML-based study showed that patient-reported questionnaires could be applicable in distinguishing headache disorders (Kwon et al., 2020). However, only a few studies have directly investigated the clinical value of predicting the efficacy of NSAIDs for acute migraine treatment (Wei et al., 2022). Moreover, a previous study showed that demographic data, migraine-related characteristics and psychiatric comorbidities were associated with the predictive power of medication overuse in migraine patients (Ferroni et al., 2020). Furthermore, some studies have reported that migraine patients with psychiatric conditions have poor response to pain treatment (Mose et al., 2018) and are prone to overuse medication (Doan et al., 2015). These ML models mainly included clinical and psychiatric characteristics or previous experience. However, very few studies have tried to identify clinical factors based on the patient-reported questionnaires that can predict the efficacy of NSAIDs for acute migraine treatment using ML methods.

In this study, we used multivariable logistic regression (LR) analysis to extract meaningful predictors from demographic, clinical and psychiatric information. Subsequently, the ML techniques based on potential predictors were performed to construct models for predicting clinical efficacy of NSAIDs in acute migraine therapy. We hypothesized that ML techniques can contribute to a better predictive model for NSAIDs efficacy. Thus, the predictive model *via* ML techniques may be vital to developing clinical decision-making strategies for migraine patients to facilitate the selection of optimal first-line treatments.

## 2 Methods

### 2.1 Participants

This was a prospective study that included a total of 673 migraine patients, who visited neurological and pain outpatient clinics between December 2017 and January 2022. All patients completed structured questionnaires during the first visit and were instructed to maintain a pain diary to record types of drugs and pain intensity before and 2 h after taking pain medication. The choice of medication was determined by the clinician’s recommendation and patient’s actual situation. Follow-up was performed at 12 weeks *via* telephone. Based on the questionnaires and clinical interview, the diagnosis of migraine was made using the International Classification of Headache Disorders third edition (ICHD-3) (Headache Classification Committee of the International Headache Society, 2018) by headache specialists with at least 5 years of experience. The exclusion criteria were as follows: 1) age <18, 2) intake of prophylactic drugs, non-NSAIDs or other long-term medications (such as anxiolytic or antidepressant drugs), 3) any other headache subtypes, 4) inability to understand the questionnaire and to comprehend the text of the informed consent form, 5) any obvious neurological conditions and cognitive impairment, 6) severe organ diseases, 7) lactation and pregnancy, and 8) incomplete data. Written informed consent was obtained from the patients.

The questionnaires primarily included demographic data (e.g., age, sex and education level), migraine characteristics (e.g., age of onset, location, family history, nausea/vomiting, photophobia, disease duration, attack duration, frequency, headache intensity, and extent of impact and burden on quality of life), and some psychiatric comorbidities (e.g., anxiety, depression and sleep disorders). Headache intensity was recorded based on the Visual Analogue Scale (VAS) with a scale from 0 to 10 (0 is no pain and 10 is the severest pain) (Aicher et al., 2012), while impact and disability on an individual were assessed by Headache Impact Test-6 item (HIT-6) (Shin et al., 2008) and Migraine Disability Assessment Scale (MIDAS) (Stewart et al., 2001), respectively. The HIT-6 questionnaire presents three questions concerning headache during the past 4-week period and a further three questions about the headache. Each of the items is scored for frequency (never = 6, rarely = 8, sometimes = 10, often = 11 and always = 13; a range of 36–78). The MIDAS score is calculated by the first five items inquiring about the number of days in the past 3 months on which migraine has an impact on the particular area of function.

Moreover, anxiety, depression and sleep conditions were measured by the Generalized Anxiety Disorder-7 item (GAD-7) (Seo and Park, 2015a), Personal Health Questionnaire-9 item (PHQ-9) (Seo and Park, 2015b) and Pittsburgh Sleep Quality Index (PSQI) (Mollayeva et al., 2016) scales, respectively. The GAD-7 contains seven questions with a minimum score of 0 and a maximum score of 21 points (each item score range of 0–3). The PHQ-9 contains nine questions with a minimum score of 0 and a maximum score of 27 points (each item score range of 0–3). The PSQI contains 19 items that are mapped onto seven components (each item score range of 0–3): 1) subjective sleep quality, 2) sleep latency, 3) sleep duration, 4) habitual sleep efficiency, 5) sleep disturbances, 6) use of sleeping medication, and 7) daytime dysfunction. The sum score of the seven components is known as total PSQI with a range of 0–21. The higher scores indicated the worse clinical symptomatology.

### 2.2 Assessment of response to therapy

The primary outcome was the reduction in the severity of pain as measured by comparing the VAS scores between before and 2 h after drug intake. Reduction in VAS scores by more than 75%, 50%–75%, 25%–50% and less than 25% were considered as complete, partial, minimal and no response, respectively. The treatment efficacy was considered effective if the reduction of headache intensity was equal to or greater than 50% with at least twice for reproducibility, and no recurrence of headache or no need to take medication again within 24 h after treatment.

### 2.3 Multivariable logistic regression analysis

Significant variables identified from the univariable analysis (*p* < 0.1) were used to perform the multivariable LR analysis for identifying the factors with the potential to predict the treatment efficacy of NSAIDs. The accuracy of the risk factor-based models in predicting response to NSAIDs therapy was assessed using receiver operating characteristic (ROC) curves. The probability value obtained from the multivariable analysis was subsequently utilized as a new input variable for the ROC curve analysis.

### 2.4 Machine learning models construction

The whole data set was randomly divided into a training/validation set (80%) and a test set (20%), and a ten-fold cross-validation strategy was used on the training/validation set for the parameter tuning of the classification models. The training/validation data were further divided into ten subsamples (nine training versus one validation). From the perspective of performance and interpretability, support vector machine (SVM), decision tree (DT) and multilayer perceptron (MLP) were finally selected. Then, the ML models were trained based on risk or all factors among demographic, migraine-related and psychiatric characteristics, respectively.

### 2.5 Statistical analysis

Continuous variables were expressed as the mean ± standard deviation (SD) and were compared between two groups using the two-sample *t*-test (for normally distributed) and Wilcoxon test (for non-normally distributed). Categorical variables were compared using the Chi-squared test or Fisher’s exact test as appropriate. Univariable and multivariable LR analyses were used to identify the potential parameters to build the prediction models. ROC curves were drawn to evaluate the models’ accuracy, measured by the area under the curve (AUC). The stratification Chi-squared test was used to compare the differences in treatment efficacy among the drugs, taking into account the differences in analgesic efficacy and safety profile of each drug. The stratification was based on independent risk factors. The results were considered significant at *p* < 0.05. All data were analyzed using SPSS version 25.0 (SPSS Inc., Chicago, IL, United States).

## 3 Results

### 3.1 Clinical characteristics

After excluding 63 patients (including 32 patients taking preventive medications) according to the predefined exclusion criteria, 610 patients were included in the final analysis. As is shown in Table 1, there were no significant differences in age, age of onset, sex, education, family history, location, attack duration, nausea/vomiting, photophobia, and HIT-6 score between the two groups. Among the characteristics screened, the migraine-related (disease duration, VAS score, frequency, and MIDAS) and psychiatric (GAD, PHQ, and PSQI scores) measurements were lower in responders compared to non-responders.

**TABLE 1:** Baseline demographic, migraine characteristics, and types of medications.

	Responders (*n* = 326)	Non-responders (*n* = 284)	*p*-value
Age (years)	35.15 ± 7.59	35.93 ± 7.37	0.150
Age of onset (years)	27.61 ± 4.99	27.16 ± 4.11	0.354
Sex (female/male)	257/69	225/59	0.921
Education (years)	12.50 ± 2.65	12.80 ± 2.68	0.135
Family history (yes/no)	91/235	96/188	0.116
Location (left/right)	174/152	146/138	0.628
Disease duration (years)	7.54 ± 5.21	8.77 ± 5.55	<0.001
VAS score	6.55 ± 3.20	7.86 ± 3.25	<0.001
Frequency (days/month)	2.76 ± 1.26	3.34 ± 1.31	<0.001
Attack duration (hours)	19.79 ± 7.72	21.73 ± 112.06	0.068
Nausea/vomiting (yes/no)	181/145	177/107	0.089
Photophobia (yes/no)	178/148	169/115	0.223
HIT-6 score	51.27 ± 4.58	51.95 ± 4.42	0.108
MIDAS score	11.76 ± 9.73	15.55 ± 13.58	<0.001
GAD-7 score	4.62 ± 2.79	5.49 ± 3.14	<0.001
PHQ-9 score	5.04 ± 3.49	6.43 ± 3.81	<0.001
PSQI score	6.60 ± 2.15	7.11 ± 2.32	0.017
Drugs	—	—	0.067
Aspirin (0.2–0.5 g/qd)	95	64	—
Ibuprofen (0.2–0.3 g/bid)	113	92	—
Acetaminophen (0.5–1.0 g/bid)	33	49	—
Naproxen (0.25–0.5 g/bid)	41	40	—
Celecoxib (0.2 g/bid)	44	39	—

GAD, Generalized Anxiety Disorder; HIT, Headache Impact Test; MIDAS, Migraine Disability Assessment Scale; PHQ, Personal Health Questionnaire; PSQI, Pittsburgh Sleep Quality Index; VAS, Visual Analogue Scale.

### 3.2 Predictive power of logistic regression and machine learning models

**FIGURE 1:**
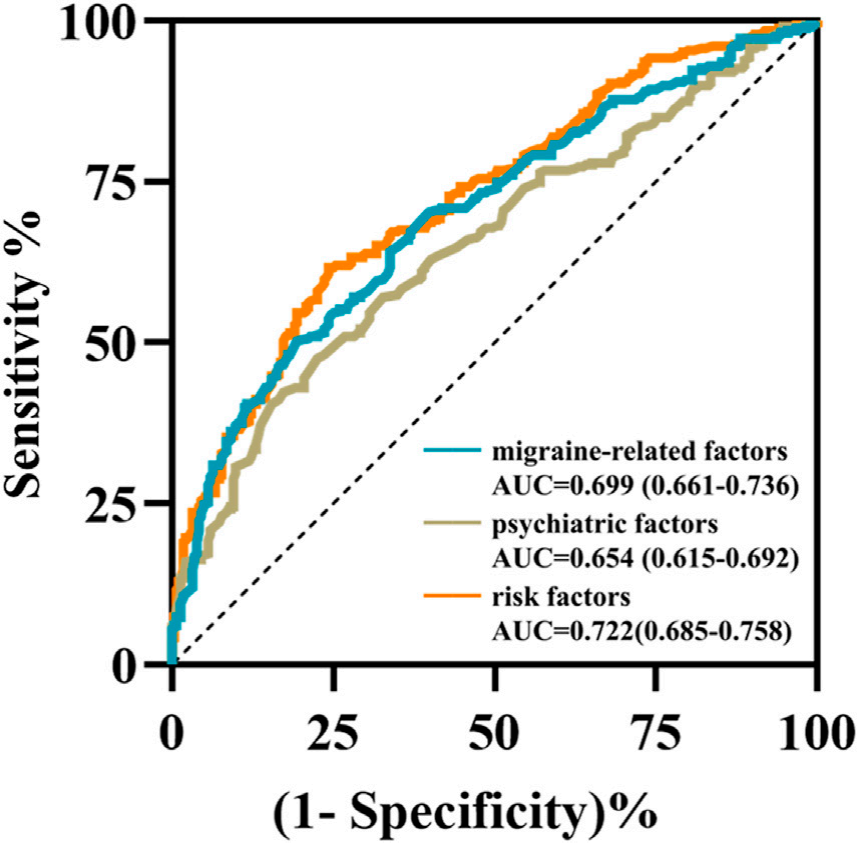
The receiver operating characteristic curves of three prediction models using multivariable logistic regression analysis. The area under the curves of model 1 (migraine-related factors), model 2 (psychiatric factors), and model 3 (six risk factors) were 0.699 (cyan curve), 0.654 (brown curve) and 0.722 (orange curve), respectively.

The multivariable LR model was conducted using variables with *p* < 0.1 between the two groups. The model showed that disease duration, headache intensity, frequency, anxiety, depression and sleep disturbance were the independent risk factors for predicting the NSAIDs efficacy (Table 2). The AUC, sensitivity and specificity values of model 1 (migraine-related factors), model 2 (psychiatric factors) and model 3 (all risk factors) were 0.699 (95% CI 0.661–0.736), 0.503 and 0.806; 0.654 (95% CI 0.615–0.692), 0.423 and 0.828; and 0.722 (95% CI 0.685–0.758), 0.617 and 0.757, respectively (Figure 1).

**TABLE 2:** Results of multivariable logistic regression analysis.

	B	Standard error	p-value	Odds ratio	95% CI
Disease duration	−0.037	0.017	0.029	0.964	0.932–0.996
VAS score	−0.343	0.060	<0.001	0.710	0.631–0.798
Frequency	−0.086	0.035	0.014	0.917	0.856–0.982
Attack duration	−0.013	0.008	0.108	0.987	0.971–1.003
MIDAS	−0.012	0.010	0.230	0.988	0.970–1.007
GAD-7 score	−0.073	0.030	0.016	0.929	0.876–0.987
PHQ-9 score	−0.054	0.026	0.035	0.947	0.901–0.996
PSQI score	−0.091	0.041	0.027	0.913	0.843–0.990

GAD, Generalized Anxiety Disorder; MIDAS, Migraine Disability Assessment Scale; PHQ, Personal Health Questionnaire; PSQI, Pittsburgh Sleep Quality Index; VAS, Visual Analogue Scale.

**FIGURE 2:**
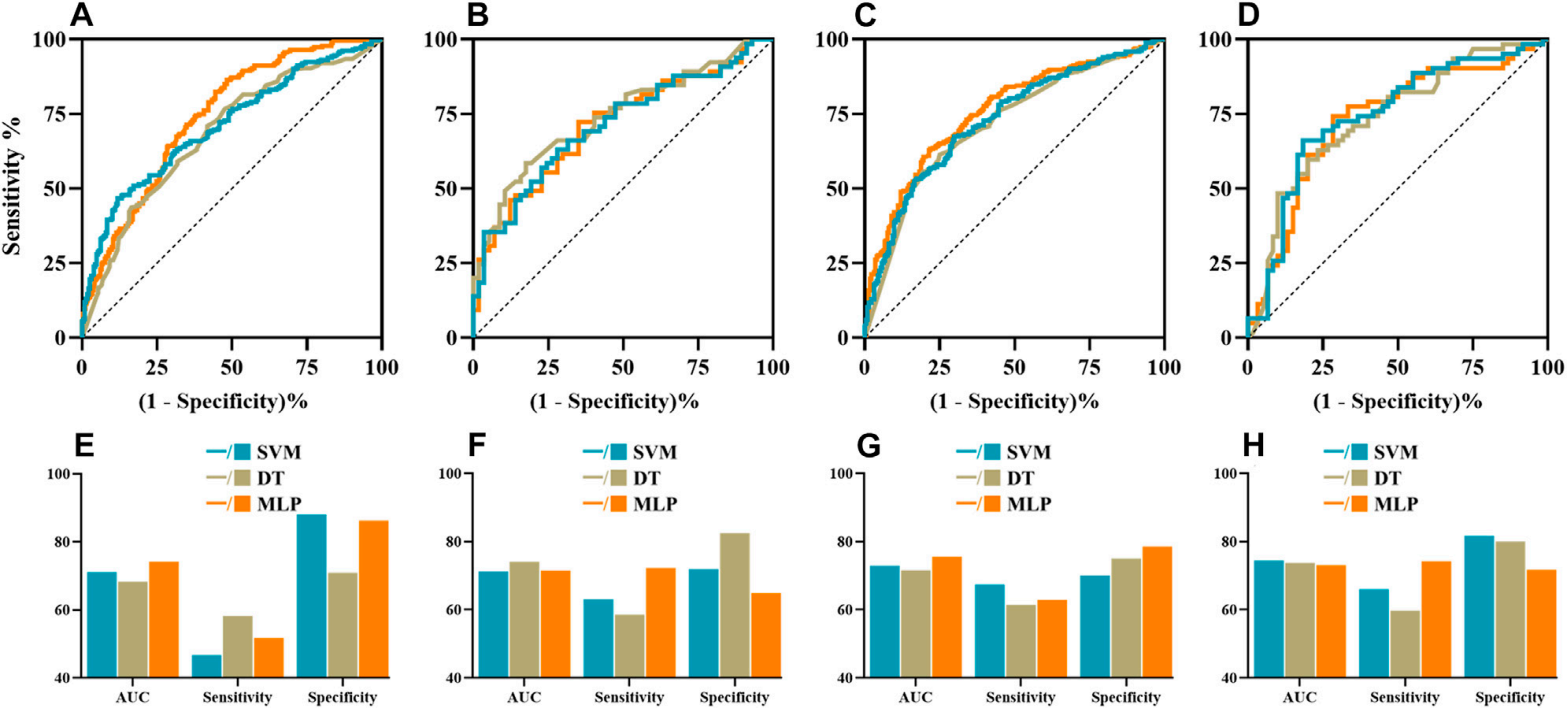
The ROC curves, AUC, sensitivity and specificity of machine learning algorithms including SVM (cyan), DT (brown) and MLP (orange). The ROC curves of ML algorithms with six risk factors **(A,B)** and all factors **(C,D)** in the validation and test datasets, respectively. The AUCs, sensitivity and specificity of ML models with six risk factors **(E,F)** and all factors **(G,H)** in the validation and test datasets, respectively. AUC, area under the curve; DT, decision tree; MLP, multilayer perception; ROC, receiver operating characteristic; SVM, support vector machine.

Then, the predictive power of ML models for NASIIDs efficacy was shown in Figure 2 and Table 3. For ML prediction models with six risk factors, the AUC, sensitivity and specificity values of SVM, DT and MPL were 0.712 (95% CI 0.623–0.791), 0.631 and 0.719; 0.741 (95% CI 0.654–0.816), 0.585 and 0.825; and 0.715 (95% CI 0.626–0.793), 0.723 and 0.649, respectively, in test cohort. For ML prediction models with all factors, the AUC, sensitivity and specificity values of SVM, DT and MPL were 0.744 (95% CI 0.657–0.818), 0.661 and 0.817; 0.737 (95% CI 0.650–0.813), 0.597 and 0.800; and 0.731 (95% CI 0.643–0.807), 0.742 and 0.717, respectively, in test cohort.

**TABLE 3:** Predictive power of machine learning models for NSAIDs efficacy.

	Risk factors (validation cohort)			Risk factors (test cohort)		
	AUC (95%CI)	sensitivity	specificity	AUC (95%CI)	sensitivity	specificity
SVC	0.711 (0.668–0.751)	0.467	0.881	0.712 (0.623–0.791)	0.631	0.719
DL	0.683 (0.640–0.724)	0.582	0.709	0.741 (0.654–0.816)	0.585	0.825
MPL	0.742 (0.700–0.780)	0.517	0.863	0.715 0.626–0.793)	0.723	0.649
	all factors (validation cohort)			all factors (test cohort)		
	AUC (95% CI)	sensitivity	specificity	AUC (95% CI)	sensitivity	specificity
SVC	0.729 (0.688–0.768)	0.674	0.701	0.744 (0.657–0.818)	0.661	0.817
DL	0.716 (0.647–0.756)	0.614	0.750	0.737 (0.650–0.813)	0.597	0.800
MPL	0.756 (0.716–0.794)	0.629	0.786	0.731 (0.643–0.807)	0.742	0.717

AUC, area under the curve; CI, confidence interval; DL, decision tree; MLP, multilayer perceptron; NSAIDs, non-steroidal anti-inflammatory drugs; SVC, support vector machine.

### 3.3 Results of stratification chi-squared tests

Independent risk factors of continuous variables were stratified and analyzed as categorical data. Univariable LR analysis was used to determine the cut-off values adjusted for the independent variables of disease duration, VAS, frequency and PSQI were 7 (years), 5 (point), 4 (days/month) and 9 (score), respectively. The cut-off values of the psychiatric variables, including anxiety (yes/no) and depression (yes/no), were determined as four points, according to the test criteria.

The Cochran-Mantel-Haenszel odds ratios (ORs) and their confidence intervals (CIs) are summarized in Supplementary Table S1. The Breslow-Day test for homogeneity of ORs across NSAIDs yielded non-significant results for all independent risk factors (except for VAS, *p* = 0.027), i.e., the distribution of ORs across NSAIDs for each risk factor is compatible with that expected given a common OR (Table 4). Furthermore, the Cochran’s test, adjusting for NSAIDs, demonstrated that there were significant independent associations between predictive factors and treatment efficacy (all *p* < 0.05) (Table 4). For variables with homogeneity of ORs, patients with more severe symptoms, including disease duration, frequency, anxiety, depression and sleep disorder, showed a decreased likelihood of response to NSAIDs with55.6%, 73.1%, 35.2%, 64.7%, and 67.8%, respectively. On the other hand, for VAS scores with inhomogeneity of ORs, patients with higher headache intensity had a decreased likelihood of response to aspirin (80.0%), ibuprofen (71.3%) and naproxen (72.9%). The variability in the efficacy of acetaminophen and celecoxib between patients with mild and severe headache was not confirmed.

**TABLE 4:** Results of Cochran–Mantel–Haenszel tests.

	Breslow-day test		Cochran’s test		Estimate	
	χ^2^	*p*-value	χ^2^	*p*-value	OR	95% CI
Disease duration	3.711	0.447	22.854	<0.001	0.444	0.317–0.622
VAS score	10.935	**0.027**	41.556	<0.001	—	—
Frequency	6.470	0.167	33.717	<0.001	0.269	0.169–0.428
Anxiety	4.025	0.403	6.934	0.008	0.648	0.468–0.897
Depression	4.352	0.360	38.366	<0.001	0.353	0.253–0.494
Sleep disorder	1.147	0.887	17.378	<0.001	0.322	0.185–0.562

CI, confidence interval; OR, odds ratio; VAS, Visual Analogue Scale. Breslow-day test: homogeneity test of OR; Cochran’s test: test of conditional independence.

## 4 Discussion

There are no standardized criteria to guide selection of NSAIDs for acute migraine therapy, and current selection is based on the trial-and-error method. The current study explored factors with the potential to predict the efficacy of NSAIDs for acute migraine therapy, and further identify if these factors affect efficacy. Multivariable LR analysis and ML models (SVM, DT and MLP) demonstrated that migraine-related (disease duration, headache severity, frequency) and psychiatric (anxiety, depression and sleep disturbance) factors had the ability to predict effective response to NSAIDs based on 12-week follow-up. Despite the fact that ML techniques had no significant benefits over multivariable LR analysis in this study, multivariable LR and ML models provide more convenient and accessible approaches to decision-making strategies with fewer characteristics and interference. Furthermore, the outcomes of this study showed that patients with more severe risk factors had a higher predisposition for decreased efficacy, suggesting an association between the risk factors and treatment efficacy of the NSAIDs. Therefore, these models for predicting NSAIDs efficacy contribute to improving drug selection at the start of treatment and avoiding unnecessary exposure of the patients to insufficient or prolonged treatment trials.

According to the evidence-based migraine guidelines (Silberstein, 2000), the major goal of migraine treatment is to relieve pain severity rapidly and prevent recurrence by reducing frequency of migraine attacks. The development of migraine is often accompanied by deteriorating clinical symptoms. As a result, the prognoses of treatment efficacy for migraine are closely related to the migraine-associated traits. Our results showed that features, such as disease duration, headache intensity and attack frequency, were associated with a decreased likelihood of response to NSAIDs. Similarly, some studies (Alpuente et al., 2020; Domínguez et al., 2018; Miller et al., 2018) demonstrated that milder headache intensity, shorter attack duration, lower headache frequency and shorter migraine duration were independent variables associated with short- or long-term response to Onabotulinumtoxin-A or occipital nerve stimulation for treating migraineurs. On the other hand, a previous study displayed contrasting results with a positive association being observed between the baseline migraine frequency and drug efficacy in patients with chronic migraine (Barbanti et al., 2021). This discrepancy in results may be due to the diversity of migraine subtypes and the difference in pathophysiological mechanisms of observed drugs. We also found that acetaminophen and celecoxib had similar efficacy between patients with mild and severe headache intensity, which was inconsistent with efficacy estimates for other drugs in the same condition. Recently, the analgesic effect of acetaminophen has been proven to be attributed to its metabolism into p-aminophenol rather than its weak inhibition of cyclooxygenase (COX) (Ohashi and Kohno, 2020). In addition, celecoxib exerts its analgesic effect by selectively inhibiting COX-2 through pharmacological mechanisms distinct from those of traditional non-selective COX inhibitors. These differences in mechanisms may not only partly account for the clinical variances among the NSAIDs, but may also reflect real differences in efficacy across different NSAIDs. Thus, the prognosis of migraine is complex and involves multiple pharmacological mechanisms, which contributes to considerable difficulty in developing an effective treatment.

It is important to point out that the criteria used for the diagnosis of migraine are based on clinical symptoms. Among the typical comorbid symptoms occurring in patients with migraine, autonomic dysfunction is frequently observed and often precedes or accompanies the onset of migraine attacks. Nausea, vomiting and photophobia are the most common and frequent dysautonomic symptoms and the major diagnostic criteria for migraine. These autonomic symptoms have been considered to be predictive factors of resistance to treatment efficacy (Takeshima et al., 2016). Gastrointestinal complaints often complicate acute migraine attacks and affect drug absorption, necessitating the use of non-oral routes of administration in patients with significant nausea or vomiting. Of note, migraine is also known as a debilitating neurovascular painful condition with strong hereditary tendency (MacGregor, 2017). A previous study (Min et al., 2013) showed a significant association between family history and autonomic dysfunction in patients with migraine, which was inconsistent with findings from our study. One possibility for this discrepancy was that a significant proportion of patients took proton pump inhibitors for symptomatic treatment of gastrointestinal symptoms. The underestimation of the impact of the gastrointestinal symptoms on our study outcomes may have obscured any potential correlation between autonomic symptoms and treatment efficacy.

This study also found that non-responders had a higher probability of psychiatric comorbidities such as anxiety, depression and sleep disturbance. Anxiety and depression are proposed risk factors for migraine chronification, while migraine headache frequency and headache severity were reported to be associated with a higher rate of anxiety and depression (Buse et al., 2019; Stubberud et al., 2021). Similar to our findings, several studies also reported that psychiatric disorders were predictive factors for assessing headache treatment efficacy (Fugger et al., 2020; Schiano Di Cola et al., 2019). As is well known, the limbic system and higher cognitive cortex are not only the core brain regions involved in regulating affective processing (Berboth and Morawetz, 2021; Kovner et al., 2019), but they are also important parts of the trigeminovascular pathway in migraine (Tolner et al., 2019). Moreover, amitriptyline, a type of antidepressant, has been recommended for prophylactic administration (Powers et al., 2013). These findings suggest an association between the pain process and emotional regulation, that is, mediated by shared underlying neural circuits, which may help explain why migraine is also comorbid with psychiatric disorders, especially depression and anxiety (Patel and Dickenson, 2022). However, our study revealed that migraineurs with psychiatric disorders had worse responses to NSAIDs relative to those without psychiatric disorders, suggesting that psychiatric comorbidities may play a crucial role in the exacerbation of migraine. Furthermore, patients with sleep-related migraine were associated with more severe and disabling clinical presentation, greater impairment of daily functioning, and greater use of symptomatic medications (Gori et al., 2015; Tiseo et al., 2020). According to these findings, identifying subtypes of migraine patients with a higher disability risk profile could have crucial implications for individually tailored management. Therefore, NSAIDs should be considered for migraine without psychiatric comorbidities, while caution should be exercised carefully when selecting pharmacological therapy for migraine with psychiatric comorbidities.

Besides, migraine-associated pain, autonomic symptoms and psychiatric comorbidities result in significant disruption of work and school as well as social and leisure activities. Migraine-induced disability and reduced quality of life, measured by the MIDAS and HIT-6 questionnaires, are the main reference indexes for assessing therapeutic efficacy (Alpuente et al., 2020; Chen et al., 2015) and the main factors driving patients to seek appropriate care (Lipton et al., 2013). Moreover, headache frequency, headache intensity and psychiatric symptoms were also associated with pain interference and headache impact (Schwedt et al., 2021). Our results revealed significant differences in migraine disability between the responders and non-responders. Although headache impact and disability are significant in the migraine process, there was no significant association observed between headache impact/disability and treatment efficacy in multivariable analysis. This discrepancy may be due to the disadvantages of patient self-reported questionnaires which may overestimate adherence or miss some patients, especially those who have minor symptoms. In addition, evidence from epidemiological studies and shared neural anatomical pathways has highlighted the importance of the relationship between clinical symptoms and treatment efficacy for acute migraine attacks. Nevertheless, this relationship is complex and affected by numerous factors. In the current study, the multivariable LR analysis and ML methods streamlined data acquisition and analysis procedure, and improved the final data quality, based on the extraction of meaningful features and elimination of interfering factors. Our results suggested the importance of a comprehensive therapeutic approach to manage headache intensity and frequency, as well as psychiatric symptoms, and optimize treatment options.

The current study has some limitations. First, the fundamental disadvantage of this study is that data were obtained in a real-life clinical population cohort, making a comparison with other therapies difficult owing to the lack of a control group. Second, we excluded those who took prophylactic and antipsychotic medications to avoid the effect on the results. However, the effect of the preventative and symptomatic treatments will require further analysis. Third, chronic migraine is highly relevant in the treatment response and prevalence of comorbidities. Given the short follow-up duration, it may misclassify some chronic migraineurs into the episodic group. So, we did not perform any analyses for this classification. However, a longer follow-up duration is required. Fourth, Medication information before the study should be noted and in relation to the response. Whether the response to the drugs prior to the study leads to milder symptoms or the milder symptoms lead to the response to the drugs should be further investigated. Fifth, these self-reported questionnaires may only show high risks rather than the diagnosis. Thus, misclassification of the outcome may have occurred and caution should be needed when interpreting results. Finally, we did not take into account the nocebo effect that negative expectations of outcomes lead to worsening symptoms. The Q-No questionnaire (Mitsikostas and Deligianni, 2015) should be considered as a potential screening tool to minimize the nocebo effect in future studies.

In conclusion, psychological and psychiatric symptoms should be carefully considered when making treatment decisions for migraine, as they play an important role in migraine development. Identifying factors that predict response to NSAIDs is important for clinicians to offer appropriate treatment strategies, optimize resources, save costs and finally improve the quality of life for the patients. Despite the fact that the ML algorithms do not outperform conventional LR analysis, this study presents a straightforward, non-invasive, reliable and reproducible method for predicting the therapeutic efficacy of NSAIDs in migraine patients. Furthermore, stratifying patients based on the risk predictors may provide rational assistance in therapeutic decision-making, resulting in a benefit from optimized individual therapy.

## Data Availability

The data analyzed in this study is subject to the following licenses/restrictions: The data of the current study would be available upon reasonable request. Requests to access these datasets should be directed to H-LW, weihengle0528@163.com.
