# Supplementary material for: Prediction and associated factors of non-steroidal anti-inflammatory drugs efficacy in migraine treatment
Source: Front Pharmacol. 2022 Nov 18;13:1002080. doi: 10.3389/fphar.2022.1002080 (PMC9754055; doi:10.3389/fphar.2022.1002080)
Supplement: Supplementary file 1 [file Table2.DOCX]

| **Supplementary Table 1** Different influence of each risk factor on treatment efficacy of different drugs. | | | | | | | |
| --- | --- | --- | --- | --- | --- | --- | --- |
|  |  | Aspirin | Ibuprofen | Acetaminophen | Naproxen | Celecoxib | Total |
| **Disease duration** | χ^2^ | 6.268 | 6.377 | 7.363 | 6.035 | 0.232 | 22.704 |
|  | *p* value | 0.015 | 0.017 | 0.011 | 0.018 | 0.663 | <0.001 |
|  | OR | 0.487 | 0.427 | 0.258 | 0.296 | 0.808 | 0.448 |
|  | 95% CI | 0.276-0.858 | 0.219-0.832 | 0.095-0.706 | 0.110-0.799 | 0.338-1.929 | 0.321-0.625 |
| **VAS** | χ^2^ | 24.469 | 12.592 | 0.172 | 6.614 | 2.765 | 42.643 |
|  | *p* value | 0.000 | 0.000 | 0.812 | 0.010 | 0.135 | <0.001 |
|  | OR | 0.200 | 0.287 | 1.222 | 0.271 | 0.423 | 0.323 |
|  | 95% CI | 0.110-0.363 | 0.142-0.581 | 0.474-3.151 | 0.097-0.754 | 0.151-1.183 | 0.229-0.456 |
| **Frequency** | χ^2^ | 7.205 | 21.738 | 5.574 | 0.739 | 3.053 | 35.655 |
|  | *p* value | 0.008 | 0.000 | 0.024 | 0.547 | 0.122 | <0.001 |
|  | OR | 0.353 | 0.084 | 0.204 | 0.589 | 0.390 | 0.259 |
|  | 95% CI | 0.162-0.771 | 0.025-0.289 | 0.050-0.838 | 0.175-1.985 | 0.133-1.143 | 0.163-0.412 |
| **Anxiety** | χ^2^ | 1.509 | 6.133 | 0.346 | 2.806 | 0.165 | 8.132 |
|  | *p* value | 0.254 | 0.020 | 0.654 | 0.116 | 0.825 | 0.005 |
|  | OR | 0.704 | 0.439 | 0.767 | 0.464 | 1.198 | 0.625 |
|  | 95% CI | 0.402-1.233 | 0.228-0.847 | 0.317-1.858 | 0.188-1.146 | 0.501-2.863 | 0.452-0.864 |
| **Depression** | χ^2^ | 22.483 | 10.594 | 1.683 | 0.698 | 6.892 | 42.062 |
|  | *p* value | 0.000 | 0.001 | 0.238 | 0.492 | 0.014 | <0.001 |
|  | OR | 0.249 | 0.341 | 0.543 | 0.680 | 0.299 | 0.337 |
|  | 95% CI | 0.139-0.448 | 0.177-0.658 | 0.215-1.373 | 0.274-1.685 | 0.119-0.748 | 0.242-0.471 |
| **Sleep disorder** | χ^2^ | 8.829 | 7.020 | 0.857 | 1.105 | 0.773 | 15.926 |
|  | *p* value | 0.004 | 0.010 | 0.465 | 0.359 | 0.504 | <0.001 |
|  | OR | 0.247 | 0.284 | 0.462 | 0.308 | 0.550 | 0.338 |
|  | 95% CI | 0.093-0.657 | 0.108-0.750 | 0.087-2.445 | 0.031-3.097 | 0.143-2.114 | 0.195-0.587 |
| CI: confidence interval; OR: odds ratio; VAS: Visual Analogue Scale. | | | | | | | |
